# Mobile phone text messaging plus motivational interviewing versus usual care: study protocol for a randomized controlled trial to evaluate effects on breastfeeding, child health, and survival outcomes, among women living with HIV (MTI-MI)

**DOI:** 10.1186/s13063-023-07647-9

**Published:** 2023-10-05

**Authors:** Moleen Zunza, Lehana Thabane, Louise Kuhn, Christine Els, Mark F. Cotton, Taryn Young

**Affiliations:** 1 Division of Epidemiology & Biostatistics, Department of Global Health, Faculty of Medicine and Health Sciences, Stellenbosch University, Francie Van Zyl Drive, PO Box 241, Cape Town, 8000 South Africa; 2 Department of Health Research Methods, Evidence, and Impact, McMaster University, Hamilton, Canada; 3 Faculty of Health Sciences, University of Johannesburg, Johannesburg, South Africa; 4 Biostatistics, Biostatistics Unit, Father Sean O’Sullivan Research Centre, St. Joseph’s Healthcare Hamilton, 3Rd Floor Martha Wing, 50 Charlton Avenue East, Hamilton, ON L8N 4A6 Canada; 5 Gertude H. Sergievsky Center, Vagelos College of Physicians and Surgeons, Columbia University Irving Medical Center, New York, USA; 6 Western Cape Department of Health, Khayelitsha District Hospital, Cape Town, South Africa; 7 Department of Paediatrics and Child Health, Family Center for Research With Ubuntu, Faculty of Medicine and Health Sciences, Stellenbosch University, Stellenbosch, South Africa

**Keywords:** Breastfeeding, HIV/AIDS, Mobile phone text messaging, Motivational interviewing

## Abstract

**Background:**

Many infants in low-resourced settings at high risk of infectious disease morbidity and death are deprived of the immunological and nutritional benefits of breast milk, through an attenuated duration of breast milk exposure. South Africa has one of the lowest exclusive breastfeeding rates in Africa, with 8% of infants under 6 months of age. We assume that breastfeeding is sustained among women living with HIV receiving weekly text messages and motivational interviewing and that this contributes to improved infant health outcomes.

**Objectives:**

(1) To evaluate the effectiveness of a combined intervention of mobile phone text messaging and motivational interviewing in promoting (a) exclusive breastfeeding and (b) any form of breastfeeding, until 6 months of child age, compared to usual care, among mothers living with HIV. (2) To evaluate the effectiveness of a combined intervention on (a) reduction in all-cause hospitalization and mortality rates and (b) improvements in infant linear growth, compared to usual care, among HIV-exposed infants aged 0–6 months.

**Methods:**

We are conducting a clinical trial to determine whether text messaging plus motivational interviewing prolongs breastfeeding and improves infant health outcomes. We are recruiting 275 women living with HIV and their HIV-exposed infants at birth and randomly assign study interventions for 6 months.

**Statistical methods:**

Breastfeeding rates are compared between the study groups using a standard proportion test and binomial regression. Survival endpoints are presented using Kaplan–Meier survival curves and compared between the study groups using the Cox proportional-hazards regression model. The count endpoint is analysed using the Poisson random-effects model and mean cumulative function. We use mixed linear regression models to assess the evolution of infant growth over time. The maximum likelihood method will be used to handle missing data.

**Discussion:**

The study findings may facilitate decision-making on (1) whether implementation of the breastfeeding policy achieved the desired outcomes, (2) interventions needed to sustain breastfeeding, and (3) whether the interventions do have an impact on child health.

**Trial registration:**

ClinicalTrials.gov NCT05063240. Pan African Clinical Trial Registry PACTR202110870407786. Oct. 1, 2021.

**Supplementary Information:**

The online version contains supplementary material available at 10.1186/s13063-023-07647-9.

## Background

Despite a consistently high antenatal human immunodeficiency virus (HIV) prevalence of about 30%, South Africa has made remarkable progress in reducing the risk of vertical transmission of HIV during the first 2 months of life, from 23% in 2003 to 0.7% in 2019 [1]. In 2016, data was published indicating the rate of cumulative vertical transmission by the age of 18 months to be at 4.3% [1]. This success is largely attributed to the adoption of the “Option B + ” strategy into policy, where mothers living with HIV receive combination antiretroviral treatment (cART), irrespective of CD4 count or clinical disease severity, to be maintained either for the duration of breastfeeding or as lifelong treatment [2, 3]. Over 90% of women living with HIV are on ART [1]. Given the benefits of breastfeeding and risks of not breastfeeding, mothers need to be supported to breastfeed their infants, while maintaining virological suppression to minimize vertical transmission risk [1]. With increased access to ART in South Africa, the national infant feeding guidelines are now the same for mothers living with and without HIV [4]. The guidelines endorse exclusive breastfeeding for the first 6 months, followed by appropriate complementary foods and continued breastfeeding for 2 years or longer [1].

Irrespective of HIV exposure, South African infants still experience high rates of morbidity and mortality [5]. South Africa’s under-five child mortality declined between 2003 and 2015, largely due to declining paediatric HIV infections and increasing availability of paediatric cART [5, 6]. South Africa’s under-five mortality rate in 2021 was at 30.8 per 1000 live births [7], double that of countries with similar-sized economies and health expenditures. HIV-exposed uninfected (HEU) children have more deaths and morbidity during infancy due to common childhood infections than HIV-unexposed infants [8, 9]. Considering the South African HIV-exposed infant population size, even a moderate additional increase in morbidity adds to the burden on the public healthcare system.

The evidence indicates that the benefits of breastfeeding outweigh the risks of not breastfeeding, regardless of the maternal HIV status [10, 11]. Breastfeeding protects against childhood mortality from diarrhoea disease and respiratory tract infections regardless of maternal HIV status [10–12]. Universal coverage with exclusive breastfeeding for 6 months and continued breastfeeding up to 1 year may prevent 13% of under-five deaths in countries with a high HIV prevalence and high under-five mortality rate [13]. Lack of breastfeeding doubles the risk of infant death in the first 6 months of life [14]. Many infants in low-resourced settings at high risk of infectious disease morbidity and death are deprived of the immunological and nutritional benefits of breast milk [15], through less breast milk exposure possibly due to efforts to reduce postnatal HIV transmission through breast milk [15, 16]. South Africa has one of the lowest exclusive breastfeeding rates in Africa, between 8 and 32% in infants under 6 months of age [10, 17, 18]. Innovative strategies are needed to encourage breastfeeding as a primary strategy for child survival [1].

The need for innovative strategies to support breastfeeding in resource-limited settings is increasingly pertinent in view of the increased disease burden on the stressed public healthcare system [1]. Interventions such as antenatal education, lactation counselling, peer counsellor support, telephonic support, and group counselling improve optimal breastfeeding practices [19]. Counselling or education delivered concurrently has a higher impact on breastfeeding rates than interventions delivered independently [19].

Patient-centred approaches for negotiating behaviour change outperform approaches that instruct patients to change behaviour through providing advice [20]. Mobile phone text messaging, a simple, low-cost intervention, improves medication adherence among patients with HIV, diabetes, and tuberculosis [21–23]. Motivational interviewing, a patient-centred non-coercive approach that explores patient readiness to change behaviour and supports the person’s commitment to do so in the preferred direction [24, 25], has been beneficial across many health problems, including HIV viral load suppression, weight loss, and alcohol and tobacco use, and medication adherence among patients with HIV and tuberculosis. Combining a number of intervention approaches is more likely to influence behaviour change than an individual approach [26].

We initiated a usual care-controlled randomized trial evaluating the effects of interactive weekly mobile phone text messaging plus motivational interviewing on sustaining breastfeeding and improving child health and survival, among mothers living with HIV. The study is an extension of our pilot study that tested the feasibility of the large trial. This group comprises 17.9% of the antenatal population in the Western Cape province, South Africa, where the majority stopped breastfeeding early [16].

## Objectives and hypotheses

The trial is primarily evaluating the effects of a combined intervention of interactive weekly mobile phone text messaging plus motivational interviewing in promoting (a) exclusive breastfeeding and (b) any form of breastfeeding, until 6 months of child age, compared to usual care, among mothers living with HIV. Secondarily, to compare the effects of the combined intervention in improving infant health outcomes, including (a) reduction in all-cause hospitalization and mortality rates and (b) improvement in infant linear growth, compared to usual care, among HIV-exposed infants aged 0–6 months. We hypothesize that breastfeeding will be sustained among mothers living with HIV receiving the combined intervention and that this will contribute to improved infant health outcomes.

## Study methods and design

### Trial design

A parallel group, adaptive [27], usual care-controlled randomized superiority trial evaluating infant feeding practices at four time points among 275 mother-infant pairs who are randomly assigned mobile phone text messaging plus motivational interviewing and usual care for 24 weeks. The fixed randomization allocation sequence was generated using blocks of different sizes, with a 1:1 allocation ratio. This trial protocol was written following the Standard Protocol Items Recommendations for Interventional Trials (SPIRIT) guidelines [28].

### Study setting

The Western Cape has a population of six million people, more than 85% rely on public health sector services and approximately 95,000 pregnant women are seen annually in public antenatal care clinics [29]. Among pregnant women living with HIV, more than 70% know their HIV-positive status before pregnancy and an estimated 90% are already on ART before pregnancy. Women receive infant feeding counselling in groups during antenatal and postnatal clinic visits. Exclusive breastfeeding at age 6 months is very low (~ 8%) [17]. Women from peri-urban informal and formal settlements are invited to participate within 24 h of giving birth at Khayelitsha District Hospital and followed for 6 months at Masiphuhlisane Research Centre in Khayelitsha.

### Eligibility criteria

Mothers living with HIV, giving birth at Khayelitsha District Hospital, are eligible if they initiate breastfeeding within 24 h of giving birth, on cART, 18 years or older, own a mobile phone, and their infants are discharged after delivery. Mothers are excluded if they initiate formula feeding within 24 h of giving birth, give birth to more than one infant, infant birthweight is < 2500 g, or gestational age is < 36 weeks. Multiple births and low birthweight are associated with suboptimal infant feeding practices and poor infant health outcomes.

### Study interventions

Irrespective of study group assignment, participants receive health services according to provincial guidelines applicable in the sector during the study. Participants are evaluated soon after giving birth and post-delivery until 24 weeks post-randomization. There are no special criteria for discontinuing or modifying allocated interventions.

### Mobile phone text messaging and motivational interviewing

Every Monday morning, a research assistant sends a text message to mothers in the intervention group, encouraging them to exclusively breastfeed and inquiring if they have any problems with breastfeeding. Mothers are requested to respond by text within 48 h, indicating whether she has a problem with breastfeeding. The research assistant contacts mothers who either indicate a breastfeeding problem or fail to respond within 48 h. Mothers have individual motivational interviews post-delivery at weeks 2, 6, and 10. During the interviews, the research assistant and the mother discuss problems with breastfeeding and potential solutions, reinforce mother’s own self-motivational statements and readiness to change, and affirm the mother’s freedom of choice. Text messaging and motivational interview are discontinued for mothers who completely stop breastfeeding but are followed until the end of the study. As soon as the mother stops breastfeeding completely, the mother-infant pair will have achieved the co-primary outcomes but will still be followed for the secondary outcomes.

### Usual care infant feeding counselling

Participants in the usual care group are counselled by primary healthcare nurses and trained lay counsellors to exclusively breastfeed for the first six months as part of routine healthcare.

### Exposure variables

Nuisance exposure variables of no primary interest: (1) timing of cART initiation: classified as prior to conception or during pregnancy according to the mother self-report or abstracted from the maternity case record; (2) viral load at delivery; (3) CD4 count at delivery; and (4) infant HIV infection status: all infant diagnostic HIV-PCR tests are performed by the national health laboratory system (NHLS). Infants are retrospectively classified at 6 months of age as HEU if there is no evidence of infant HIV infection in the NHLS database or as HIV infected; mother’s HIV disclosure status: classified as yes or no according to mother self-report; baseline socio-demographic characteristic obtained by interviewing the mother at study entry.

### Endpoint variables

Exclusively breastfeeding is defined as a child has only breastmilk and no other liquids or solid foods until 6 months of age. Any form of breastfeeding is defined as the child has breastmilk and other liquids or solid foods until 6 months of age. Infant feeding practices are assessed using a questionnaire of food items given to the baby in the last week or 24 h preceding the inquiry [30]. The age of child at the time of stopping breastfeeding is obtained by interviewing the mother. Infant death or hospitalization for any cause occurring within the study duration is identified by interviewing the mother. Non-routine or sick-clinic visits as reported by the mother. Infant weight and length are measured by the research assistants at study visits.

### Safety events

Safety events are collected from the time the participant consents to trial participation until the participant is discharged from the trial or at the trial conclusion. Potential social unintended consequences of safety events include relationship conflicts with partners due to study participation; reduced child monitoring due to exaggerated perception of the benefits of breastfeeding (assess infant immunization history as proxy), psychological stress, or guilt due to non-compliance with exclusive breastfeeding; and inadvertent disclosure of participant’s HIV status.

### Sample size, power, and detectable differences

We expect an exclusive breastfeeding rate of 8% at age 6 months in the usual care group [17]. We want to detect a difference of 15% between the intervention and usual care groups (i.e., 23% vs 8%). The traditional fixed sample size computations using WinPepi give a sample size of 182 for a two-sided test. Using inflation factor 1.01 for two planned analyses of O’Brien-Fleming boundaries at the 0.05 significance level and 80% power, we compute the maximum sample size of 182 × 1.01 = 184. The sample size is inflated by 33% to 275, to account for loss to follow-up (30%) and infants who test HIV positive (3%) who are included in the analysis ignoring infant HIV status and repeat the analysis excluding HIV-infected infants. The study is not powered for the co-primary endpoint (i.e., any form of breastfeeding rate at 6 months of age) and secondary endpoints. The co-primary and secondary endpoint analyses are considered exploratory and may only be supportive of the primary analysis of exclusive breastfeeding endpoint. No adjustment to the significance level for testing of multiple co-primary endpoints is made since all the co-primary endpoint analyses are considered exploratory.

### Recruitment and retention

Enrolment started at Khayelitsha District Hospital on 22 July 2022. We aim to enrol 275 mother-infant dyads by the end of 2023. Informed consent is requested to conduct an enrolment maternal interview; four in-person follow-up visits at weeks 2, 6, 10, and 24 post-randomization; child hospitalization medical record review; and child height and weight measurements (Table 1). Eligible mothers are consented to the study by a research assistant in the mother’s language of her choice. Mothers are informed of the risk of loss of confidentiality and the need to access medical records. Mothers sign the informed consent form in a private space at the hospital. The research assistant conducts an enrolment maternal interview and completes a baseline questionnaire. The study provides transport to and from the study follow-up research site; mothers receive a R180 (~ US $10) voucher at each study visit, for their participation time and any other study-related costs. A participant who considers withdrawing from the study is informed about modified follow-up options (telephone interviews). If a participant withdraws from the study, we confirm if the participant agrees to medical and laboratory records access in line with the original consent. For a participant who fails to return for a study visit, the research team attempts to contact the participant and reschedule the missed visit. Before a participant is deemed lost to follow-up, efforts to regain contact with the participant continue through telephone calls or home visits.

**Table 1:** Time schedule of enrolment, interventions, assessments, and visits for participants

	**Recruitment**	**Visit1**	**Visit 2**	**Visit 3**	**Visit 4**	**Study completion form**
**Target**	At delivery	2 weeks	6 weeks	10 weeks	24 weeks	To be completed when the participant is LTFU, withdraws from the study, or at the end of the study
**Window**	Within 24 h of giving birth	± 4 days	± 7 days	± 14 days	± 14 days	
**Screening for eligibility**	x					
**Detailed informed consent**	x					
**Randomization**	x					
**Visit preparation, follow-up, and tracking of participants**						
Telephonic reminder of a visit		x	x	x	x	
Schedule next visit and complete appointment card	x	x	x	x		
**Interventions**						
Intervention group: text messaging weekly for 24 weeks						
Intervention group: motivational interview		x	x	x		
Usual care group: referred for usual care if necessary		x	x	x		
**Assessment**						
Demographics	x					
Baseline data	x					
Anthropometrics		x	x	x	x	
Maternal NHLS antenatal CD4 count and viral load	x					
NHLS infant HIV-PCR result				x	x	
Infant feeding questionnaire		x	x	x	x	
Health questionnaire including medications (e.g. immunization, morbidity and mortality, infant weight, and height measurements)		x	x	x	x	
Health record reviews—hospital electronic administration systems: infant hospitalization and mortality					x	

### Sequence generation and allocation concealment mechanism

The principal investigator used Stata 17 random number generating command (RALLOC), to generate the fixed randomization allocation sequence using permuted block sizes of 2, 4, and 6. Participants are enrolled by the research assistant. Baseline data are captured using the Research Electronic Data Capture (REDCap) mobile application and uploaded to the REDCap database. The principal investigator assigns a study group sequentially, using the allocation table that is locked, unmodifiable and stored in the REDCap database.

### Blinding

A research assistant not administering the study interventions completes study questionnaires at follow-up visits, without knowledge of the study assignment. A research assistant administering the study interventions is not blinded to the study assignment. The independent data analyst performs unblinded analyses. Study participants are aware of the assigned study group.

### Study procedures and measurements

**Fig. 1:**
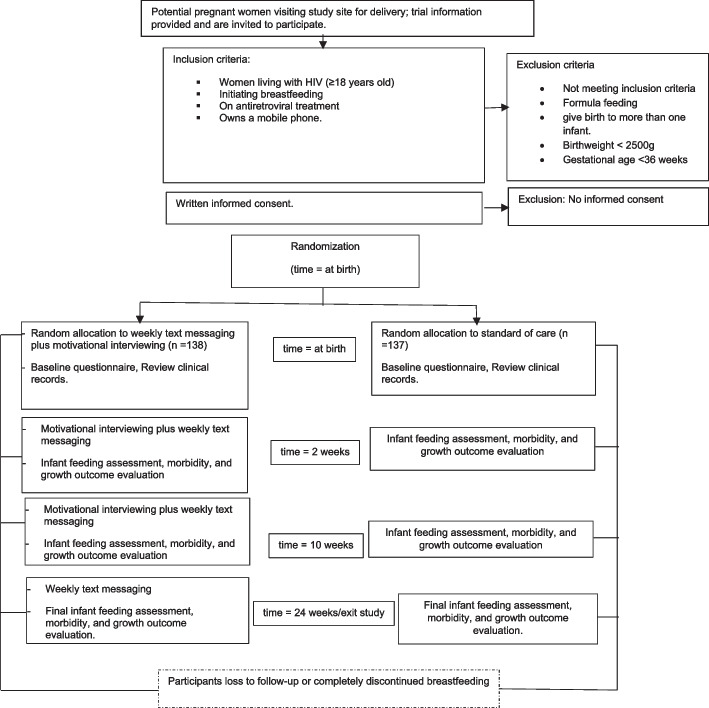
Study participant flow diagram: schedule of enrolment, interventions, and assessments

Baseline sociodemographic and clinical characteristics are obtained by interviewing the mother and abstracting medical records data. Following enrolment at Khayelitsha District Hospital, participants have in-person follow-up visits at Masiphuhlisane Research Centre. At each visit, a research assistant interviews the mother using a questionnaire of food items given to the baby in the last week or 24 h preceding the inquiry. The infant feeding questionnaire was developed using the World Health Organization standardized instrument [30]. Infant death, hospitalization, and safety events are identified by interviewing the mother. Infant weight and height are measured at each visit. Participants who fail to attend a visit are interviewed telephonically. See the study participant flow diagram in Fig. 1.

### Data management

Data are entered remotely using password-protected tablets and stored electronically on password-protected REDCap database hosted at Stellenbosch University institutional servers. The principal investigator (PI) manages the REDCap database access. Research assistants are granted permission for data entry. The data analyst has permission to view the database and will only receive anonymized de-identified datasets at the time of analysis. The PI reviews data quality and can make edits to the database. Study documents, including signed informed consent forms, are filed in a lockable cabinet at study sites. After database lock, all electronic case report forms are archived for 15 years. The data will be deposited into the SUNScholarData, a data repository that is managed by the Stellenbosch University Library.

### Statistical methods

The participant is the unit of reporting. The interim analysis includes the first 138 mother-infant pairs enrolled (half of the total enrolment). Table 2 provides a summary of methods of analysis for each endpoint. An independent data analyst performs data analysis during trial monitoring. The data analyst writes a statistical code that includes the population used in a table or figure that is explicitly set at the start of a block of code that computes the output; at the start of any code, a set of comments give references to inputs and outputs. The Stata software package is used for analysis.

**Table 2:** Endpoints, hypothesis, and methods of analysis

Endpoint measure	Hypothesis	Method of analysis
Primary 1: exclusive breastfeeding at 6 months of child age	Text messaging and motivational interviewing lead to higher rates of exclusive breastfeeding than usual care	▪ Standard proportions test ▪ Binomial regression
Primary 2: any form of breastfeeding at 6 months of child age	Text messaging and motivational interviewing lead to higher rates of any form of breastfeeding than usual care	▪ Standard proportions test ▪ Binomial regression
Exploratory analysis of primary endpoint: time to stopping breastfeeding	Text messaging and motivational interviewing lead to a higher median time to stopping breastfeeding than usual care	▪ Kaplan–Meier survival analysis ▪ Cox proportional-hazards regression model
Secondary 1: number of all-cause infant hospitalization and death as a composite endpoint Time to first all-cause death or hospitalization	Text messaging and motivational interviewing lead to a reduction in the number of all-cause hospitalizations or all-cause deaths than usual care	▪ Mean cumulative function ▪ Poisson random-effects model ▪ Kaplan–Meier survival analysis ▪ Cox proportional-hazards model
Secondary 2: infant growth	Text messaging and motivational interviewing lead to improved infant growth than usual care	▪ Mixed linear regression models

### Primary efficacy confirmatory analysis

A group-sequential standard proportion test using the O’Brien-Fleming boundaries rejects the null hypothesis of no difference in exclusive breastfeeding rates between the intervention and usual care groups, at the end of the study if the computed *z*-statistic exceeds the prespecified stopping boundary value of 1.9776.

### Exploratory efficacy analyses of primary endpoints

The potential confounding effect of baseline sociodemographic and clinical variables is adjusted for in the binomial regression, Cox proportional hazards, mixed linear regression, and Poisson regression models.

#### Dichotomous endpoints

To determine whether text messaging plus motivational interviewing leads to higher rates of (i) exclusive breastfeeding or (ii) any form of breastfeeding than usual care, the relative risk (by binomial regression) of exclusive breastfeeding or any form of breastfeeding is computed for the study group.

#### Time to event endpoints

Analysis of time to stopping breastfeeding is presented by Kaplan–Meier survival curves separately by study group and comparisons are made with the log-rank test. Participants are censored at 24 weeks or the last completed visit. A Cox proportional hazard regression analysis on the study group, with stopping breastfeeding as the endpoint, using the age of child at the time of stopping breastfeeding as the time at risk of the hazard of stopping breastfeeding is compared to binomial regression for consistency of associations.

### Exploratory efficacy analyses of secondary endpoints

#### Time to event endpoints

Time to first all-cause death or hospitalization is analysed using Kaplan–Meier survival curves and Cox proportional-hazards model. Time from birth to occurrence of first hospitalization or death is used as time at risk for the survival analysis.

#### Continuous endpoints

The evolution of weight-for-age, length-for-age, and weight-for-length *z*-scores (World Health Organization standardized) is compared between the study groups using mixed linear regression models.

#### Count outcomes

A Poisson random-effects model is used to compare counts of all-cause infant hospitalization, death, and non-routine sick clinic visits between the study groups, allowing for a lack of statistical independence resulting from the observation of multiple events in some infants. Results are adjusted for calendar time to account for infant health progression, leading to a decreasing incidence of events among infants over time.

The number of all-cause infant hospitalization, death, and non-routine or sick clinic visits (composite endpoint) is compared between the study groups using the mean cumulative function. The intensity rate is reported.

### Safety analyses

Safety data are summarized as count (percent) by study group and in aggregate. When calculating the incidence of safety events, by study group, study visit, and overall, each participant is counted once, and any repetitions are ignored; the denominator is the total sample size.

### Sub-group analyses

Exploratory sub-group analyses are conducted to assess the consistency of intervention effect across potential prognostic factors. Subgroup analyses focus on the evidence for a difference in intervention effects on the primary endpoints: the interaction effect. We present exploratory subgroup-specific summary statistics and use forest plot figures showing possible subgroup effects and interactions.

If the sample size permits, we create the following subgroups and test our hypotheses on the following:

- Subgroup 1: subdivide the population into two baseline viral load strata—suppressed viral load and high viral load
- Analysis of participants with different follow-up times: complete 1 study visit, 2 study visits, 3 study visits, and all 4 study visits to determine the incremental benefit gained by each additional motivational interview

### Missing data

Infant weight and height measurements and feeding practices will be missing for participants who are lost to follow-up, who miss study visits and with premature discontinuation from the study. The missing values may depend on observed data and will consider these missing values as missing at random. If there is no correlation between the missing values and the observed data, i.e., Little’s test is not statistically significant (*p* > 0.05), missing values for the outcome variables will be considered missing completely at random. We specify the analysis model, for estimating the intervention effect on stopping breastfeeding, and evaluate how that effect may have changed over the 24-week period and specify maximum likelihood as the method to handle missing data. A multivariate normal model will be specified for the relationships among variables that have missing data. We fit a mixed (random intercept) model with a log link and unstructured covariance matrix or Toeplitz covariance matrix to deal with missing data on repeated measures. Those who drop out contribute records for those time points in which they respond, but not for the later time points. If missing values are missing completely at random or missingness is confined to the outcome variable, we use complete case sensitivity analysis for our primary analyses. Infant growth is estimated and compared between study groups using mixed linear regression models with maximum likelihood estimation for dealing with missing data under the missing at random assumption.

### Planned schedule of interim analysis

One interim analysis meeting is held to review data on intervention efficacy and safety. The Data Safety Monitoring Board (DSMB) assess between groups (intervention vs usual care): (i) preliminary assessments of efficacy (i.e., exclusive breastfeeding rate) and ii) co-primary endpoint, any form of breastfeeding rate and secondary endpoints.

Interim analysis is performed after 69 participants per group have complete response data. A group-sequential test using the O’Brien-Fleming boundaries rejects the null hypothesis of no difference in exclusive breastfeeding rate between the intervention and usual care groups, at the interim analysis if the computed z-statistic exceeds the O’Brien-Fleming boundary value of 2.7967. The DSMB determine whether this predefined trial efficacy stopping rule is met.

### Stopping rules

The DSMB members determine whether predefined trial efficacy stopping rules is met, the DSMB makes a recommendation to the study team to continue or to terminate the trial.

### Monitoring

#### Data monitoring

The trial steering group meets bi-monthly reviewing trial conduct and progress. The DSMB has six voting members with expertise in HIV and infant feeding, epidemiology, and randomized clinical trials. The DSMB membership is restricted to individuals without significant conflicts of interest who independently oversee and evaluate the efficacy of the interventions, safety, study conduct, and scientific validity and data integrity of the study at yearly intervals and ad hoc, if necessary, until the last participant completes the study. The DSMB provides a recommendation to investigators, whether the study should continue unchanged, be stopped due to adverse effects or efficacy, or be modified in any way.

### Trial safety monitoring

The DSMB reviews the unblinded safety data accruing in the study, including an assessment of relatedness to trial intervention. Adverse events or unanticipated problems involving risks to participants are reported to DSMB, EDCTP project officer, and ethics committee within seven working days of the event, including a description of the measures taken in response to the event (if any). The DSMB can suspend the trial for safety concerns even if no formal holding rule has been predefined and recommend appropriate action.

### Procedure if the trial is put on hold

If the trial is put on hold because of a safety concern, enrolment in the trial and all interventions cease immediately, but safety procedures continue. The DSMB reviews all available safety data and makes a recommendation to the study team whether the trial should be stopped permanently, modified, or continue unchanged.

### Quality assurance of statistical programming

At the end of the trial, the study team will independently conduct the primary analyses and inform the DSMB, to ensure concordance between what the DSMB has reviewed during study monitoring. The study team review the entire analyses and check the programming code used by the data analyst.

### Ethical considerations

Stellenbosch University Human Research Ethics Committee (reference M21/03/010) approved the study. Western Cape Department of Health approved access to Khayelitsha District Hospital for study recruitment (reference WC_202107_007). Study participation is voluntary, mothers have the right to withdraw their participation at any time. Mothers undergo an informed consent process with a research assistant, in their native language Xhosa or English and sign a written informed consent document for their and their child’s participation in this study. In the unlikely event of the participant sustaining physical injury through study participation, the participant is insured by Stellenbosch University’s research policy.

Mothers have the option to consent or decline to their and their child’s anonymized and de-identified data being contributed to SUNScholarData and pooled with other maternal-child studies with appropriate institutional review board approvals for any additional pooled analyses. As children are enrolled and followed to age 6 months, no assent is feasible. The ethics committee have access to source data documents for monitoring purposes. A progress report is submitted annually, for renewal of ethics approval by the Stellenbosch University Human Research Ethics Committee. The data analyst has access to the anonymized final dataset. The trial was registered on ClinicalTrials.gov (NCT05063240) and Pan African Clinical Trial Registries (PACTR202110870407786) before the recruitment of the first subject.

## Discussion

Our study complements other work done in our setting and is aligned with the South African national and provincial sustainable development priorities. The work presents an opportunity to determine whether text messaging and motivational interviewing sustain breastfeeding and improve infant health outcomes. Thus, this project contributes towards achieving the Sustainable Development Goal 3 call for an end to preventable deaths of children under 5 years. Findings from this study may facilitate decision-making on (1) whether implementation of the breastfeeding policy achieved the desired outcomes, (2) interventions needed to sustain breastfeeding among women living with HIV, and (3) whether the interventions do have an impact on child health and survival. Should we find no effect of text messaging and motivational interviewing on sustaining breastfeeding and improving child health, the study can still inform decision-making regarding breastfeeding policy. Should we find the intervention effective, we will, in collaboration with the Western Cape Provincial Vertical Transmission Prevention of HIV strategic programme adapt the health services practices to provide the necessary support to sustain breastfeeding for longer.

## Trial status

Protocol version number 1 31 May 2021, recruitment began on 22 July 2023, and the approximate date recruitment is expected to be completed is 31 October 2023.

### Supplementary Information

**Additional file 1. **

## Data Availability

The data will be deposited into the SUNScholarData, a data repository that is managed by the Stellenbosch University Library. Mothers have the option to consent or decline to their and their child’s anonymized and de-identified data being contributed to SUNScholarData and pooled with other maternal-child studies with appropriate institutional review board approvals for any additional pooled analyses.

## Abbreviations

cART: Combination antiretroviral treatment
DSMB: Data Safety Monitoring Board
EDCTP: European & Developing Countries Clinical Trials Partnership
HEU: HIV-exposed uninfected
HIV: Human immunodeficiency virus
NHLS: National Health Laboratory System
PI: Principal investigator
REDCap: Research Electronic Data Capture
